# Immunophenotypic Features of CD68 and CD163 Distribution in Oral Squamous Cell Carcinoma of Tongue and Lip

**DOI:** 10.3390/diseases14100363

**Published:** 2026-10-02

**Authors:** Grigory Demyashkin, Andrey Kostin, Maya Epifanova, Alexandr Epifanov, Ekaterina Blinova, Anton Kasianov, Vladimir Shchekin, Dibakhan Tsomartova, Matvey Vadyukhin, Petr Shegai, Andrei Kaprin

**Affiliations:** 1 Department of Digital oncomorphology, National Medical Research Centre of Radiology, 2nd Botkinsky Passage, 3, 125284 Moscow, Russiavma20@mail.ru (M.V.);; 2 I.M. Sechenov First Moscow State Medical University ( Sechenov University), Trubetskaya Street, 8/2, 119048 Moscow, Russia; 3 Department of Urology and Operative Nephrology with Oncourology Course of the Medical Institute, Peoples’ Friendship University of Russia Named After Patrice Lumumba, Miklukho-Maklaya Street 6, 117198 Moscow, Russia; 4 Research and Educational Resource Center for Immunophenotyping, Digital Spatial Profiling and Ultrastructural Analysis Innovative Technologies, Peoples’ Friendship University of Russia Named After Patrice Lumumba, Miklukho-Maklaya Street 6, 117198 Moscow, Russia; 5 Department of Fundamental Medicine, Institute for Physics and Engineering in Biomedicine, National Research Nuclear University MEPhI, Kashirskoe Shosse 31, 115409 Moscow, Russia

**Keywords:** oral squamosis cell carcinoma, OSCC, tumor microenvironment, tongue, lip, CD68, CD163, macrophages

## Abstract

Background and Objectives: Oral squamous cell carcinoma (OSCC) belongs to a broad group of malignant neoplasms of the head and neck. It is known that components of the tumor microenvironment (TME) influence tumor progression. Similar patterns are being actively investigated in OSCC. In particular, a high density of CD68^+^ macrophages is associated with longer survival, whereas an abundance of M2 macrophages (CD163^+^) correlates with metastasis and an unfavorable prognosis. The present study compares the density and spatial distribution of CD68^+^ and CD163^+^ macrophages between intratumoral and peritumoral compartments in OSCC of the tongue and lip. Materials and Methods: Data from 112 patients with a verified OSCC diagnosis were included in this study. For the comparative morphological analysis, two core groups were formed: (a) Group I (*n* = 84), patients with Squamous Cell Carcinoma (pT1) of the tongue; (b) Group II (*n* = 28), patients with Squamous Cell Carcinoma (pT1) of the lip. Immunohistochemistry was performed according to standard protocols. Results: Tongue OSCC was characterized by the predominant accumulation of both CD68^+^ and CD163^+^ macrophages in the peritumoral stroma compared with the intratumoral zones (34.2 ± 8.1 vs. 48.1 ± 6.4 cells/mm^2^). In contrast, in lip OSCC, a higher density of CD68^+^ macrophages was observed in the intratumoral compartment, whereas the number of CD163^+^ macrophages remained considerably lower in both the intratumoral (51.1 ± 4.3 vs. 9.4 ± 2.1 cells/mm^2^) and peritumoral stroma of the tumor. Conclusions: These findings demonstrate that tongue and lip OSCC harbor distinct patterns of macrophage distribution and polarization, underscoring the role of the immune microenvironment in the biological heterogeneity of OSCC and warranting further investigation into macrophage subtype-specific prognostic markers.

## 1. Introduction

Oral squamous cell carcinoma (OSCC) belongs to a broad group of malignant neoplasms of the head and neck [1]. According to the WHO classification, OSCC is histologically divided into keratinizing and non-keratinizing types, which differ in the degree of differentiation, morphology, and localization [2]. The most common sites of OSCC are the tongue and the lip. Tongue cancer is typically represented by poorly differentiated forms of the non-keratinizing type, characterized by aggressive growth and early metastasis, thereby contributing significantly to overall cancer mortality, which accounts for hundreds of thousands of cases annually according to GLOBOCAN [3]. In contrast, lip cancer is more often a well-differentiated keratinizing tumor (G1) with pronounced keratinization; it is associated with chronic sun exposure and characterized by a more indolent clinical course and a better response to treatment [4]. However, the overall prognosis for OSCC is unfavorable when detected at advanced stages, and the 5-year survival rate rarely exceeds 50–60%, underscoring the need to identify novel prognostic and predictive biomarkers, including immunohistochemical ones [5]. Furthermore, comprehensive evaluation and early identification of disease- and treatment-related functional impairment, such as oral and nutritional dysfunction, have been shown to be critical for optimizing patient management and long-term outcomes in OSCC [6].

It is known that components of the tumor microenvironment (TME) influence tumor progression [7]. The cellular composition of the TME is heterogeneous and includes immune (lymphocytes, macrophages), stromal (fibroblasts), and vascular components that interact with one another, with tumor cells, and with elements of the extracellular matrix [8]. Thus, the significant impact of the immune component of the TME on tumor growth and prognosis has been convincingly demonstrated in triple-negative breast cancer, where the level of lymphocyte infiltration is an independent prognostic factor [9]. Similar patterns are being actively investigated in OSCC. In particular, a high density of CD68^+^ macrophages is associated with longer survival, whereas an abundance of M2 macrophages (CD163^+^) correlates with metastasis and an unfavorable prognosis [10].

Therefore, it is conceivable that the density and spatial distribution of immune cell subpopulations within the TME in OSCC could be regarded as prognostic biomarkers [11]. In this regard, current research approaches place particular emphasis on studying the spatial organization of the immune infiltrate. In OSCC, not only the density but also the spatial interactions of macrophages in different tumor compartments hold key prognostic significance, as they reflect the balance between the antitumor immune response and immunosuppression [12].

It is well established that macrophage counts are highest precisely at the earliest stage of tumor development; accordingly, the present study will focus specifically on the pT1 stage [13].

This study aims to conduct a comprehensive histological and immunohistochemical comparative assessment of the influence exerted by key microenvironmental elements—namely, CD68^+^ and CD163^+^ macrophages between intratumoral and peritumoral compartments in OSCC of the tongue and lip. Importantly, OSCCs will be taken into account at the pT1 stage. This will make it possible to capture the dynamics of immunosuppressive and pro-tumoral niche formation at an early phase of tumor progression.

## 2. Materials and Methods

### 2.1. Patients

The present work is an observational, analytical cohort study of retrospective, multicentre design, performed on the clinical basis of the P. Hertsen Moscow Oncology Research Institute (Moscow, Russian Federation). Eligible cases were retrieved by querying the electronic medical databases of the participating centres for entries coded under the 10th revision of the International Classification of Diseases (ICD-10) as C00 (malignant neoplasm of the lip) or C02 (malignant neoplasm of other and unspecified parts of the tongue).

Data from 112 patients with a verified OSCC diagnosis were included in this study. The study was approved by the Ethics Committee of the RUDN University named after Patrice Lumumba (Protocol No. 3 dated 10 March 2026), and also complied with the requirements of the Declaration of Helsinki (WMA Declaration of Helsinki—Ethical Principles for Medical Research Involving Human Subjects, 64th WMA General Assembly, Fortaleza, Brazil, October 2013). The analysis relied predominantly on medical records, specifically patient case histories. Key elements reviewed for all individuals comprised their anamnesis, clinical symptoms, tumor stage (assessed via the AJCC 8th edition classification system), details of any surgical interventions and non-surgical therapies administered, as well as final disease outcomes. Furthermore, an examination of surgical pathology samples (paraffin-embedded tissue blocks) stained with hematoxylin and eosin was conducted to evaluate the histological subtypes of OSCC. For the comparative morphological analysis, two core groups were formed:

- Group I (*n* = 84): Patients with Squamous Cell Carcinoma (pT1) of tongue;

- Group II (*n* = 28): Patients with Squamous Cell Carcinoma (pT1) of lip.

In the presence of squamous cell carcinoma of the oral cavity, the diagnosis was confirmed by a comprehensive laboratory and instrumental examination, including an excisional or incisional biopsy and subsequent histological verification. To assess the local extent of the tumor and stage it according to the TNM classification, imaging techniques were used: multislice computed tomography (MSCT) and/or magnetic resonance imaging (MRI), and, when necessary, positron emission tomography (PET) of the head and neck. The diagnosis was confirmed based on a combination of comprehensive morphological and radiological data.

Inclusion criteria: the histological type of tumors was determined in accordance with the criteria of the 5th edition of the World Health Organization Classification of Head and Neck Tumors (2022) [2]; squamous cell carcinoma of the tongue and lip; the stage of the malignant neoplasm corresponded to Stage I. Staging was performed according to the 8th edition of the TNM classification of the American Joint Committee on Cancer (AJCC); the functional status of patients according to the ECOG scale corresponded to 0–1 point.

Exclusion criteria: tumor size greater than 20 mm (T2–4), presence of distant metastases (M1) and metastases to regional lymph nodes (N1–3) at the time of primary diagnosis (Stage II–IV AJCC); previously registered recurrence of the disease or any type of antitumor treatment (surgical, drug, or radiation) for this disease prior to inclusion in the study; presence of synchronous or metachronous primary multiple malignant neoplasms of other localizations; nonepithelial tumors of the oral cavity (mesenchymal, neural, etc.); the presence of severe decompensated comorbidities (including infectious and autoimmune diseases) that could significantly affect overall survival and the possibility of specific antitumor treatment; insufficient clinical data or unsuitability of histological material for research.

### 2.2. Morphological Block

#### 2.2.1. Histologic Research

Serial 2 μm sections were cut from archived, formalin-fixed paraffin-embedded tumour blocks and routinely stained with haematoxylin and eosin. Slides were examined under a Leica DM2000 photomicroscope (Leica Microsystems, Wetzlar, Germany) following conventional histopathological criteria. Histological subtyping was performed according to WHO classification (2022) [2] based on the presence/absence of keratin pearls and intercellular bridges.

#### 2.2.2. Immunohistochemical (IHC) Assay

Sections underwent routine immunohistochemical processing with the primary antibodies anti-CD68 (Abcam, ab303565; Cambridge, UK; 1:1000) and anti-CD163 (Abcam, ab182422; Cambridge, UK; 1:500). Bound primary antibody was visualised with the two-step HiDef Detection™ HRP polymer kit (Cell Marque, Rocklin, CA, USA), comprising an anti-mouse/anti-rabbit IgG linker, horseradish-peroxidase label and DAB chromogen; nuclei were counterstained with Mayer’s haematoxylin. Splenic tissue, a site of dense macrophage accumulation, served as the positive control for CD68, and tonsillar tissue (likewise rich in macrophages) for CD163. For negative controls, serial sections were processed identically but with the primary antibody omitted and replaced by isotype-matched serum, allowing evaluation of nonspecific background and chromogen artefact.

For each case, representative regions of interest were selected within viable OSCC tissue, avoiding areas of extensive hemorrhage, necrosis, tissue folds, cautery artifact, and non-representative section edges. When stromal remodeling was present, regions with hyalinosis or fibrosis were analyzed separately or recorded as a morphological variable. Slides were digitised on a Leica Aperio AT2 scanner (Leica Microsystems, Wetzlar, Germany), and immunopositive cell counts were extracted from the resulting whole-slide images. Cell density (cells/mm^2^) and absolute counts were obtained by parametric quantification in QuPath (v0.5.1), an open-source platform for digital pathology. The spatial distribution of CD68^+^ and CD163^+^ macrophages was mapped using QuPath’s native density-mapping tool, which applies Gaussian-kernel smoothing to generate heat-map overlays.

All variables were abstracted from the electronic medical-record system into a password-protected Microsoft Excel 2010 database (Microsoft Corporation, Seattle, WA, USA). Two independent co-investigators cross-checked every entry against the source records, and discrepancies were resolved by consensus prior to statistical analysis; this dual-verification step was intended to limit transcription error and safeguard data integrity.

### 2.3. Statistical Significance

All computations were carried out in SPSS v12.0 (IBM Analytics, USA). Distribution normality was checked with the Shapiro–Wilk test, with *p* > 0.05 taken to indicate a Gaussian distribution; since this assumption was not met, between-group comparisons of continuous variables relied on the non-parametric Mann–Whitney U test, with *p* < 0.05 taken as the significance threshold. Tabulated values are reported as medians with interquartile ranges (Me [Q1–Q3]), and violin plots were generated with BioRender.com (https://www.biorender.com; accessed on 12 August 2026) to illustrate group distributions.

## 3. Results

### 3.1. Characteristics of Patients

Data and patient characteristics are presented in Table 1. The study included 112 patients, with a mean age of 64.2 ± 6.8 years. Two types of OSCC were considered: malignant neoplasm of lip, with 28 patients, and malignant neoplasm of other and unspecified parts of tongue, with 84 patients. According to the AJCC (8th edition) TNM classification, all tumors were classified as pT1.

### 3.2. Histological Patterns

**Figure 1:**
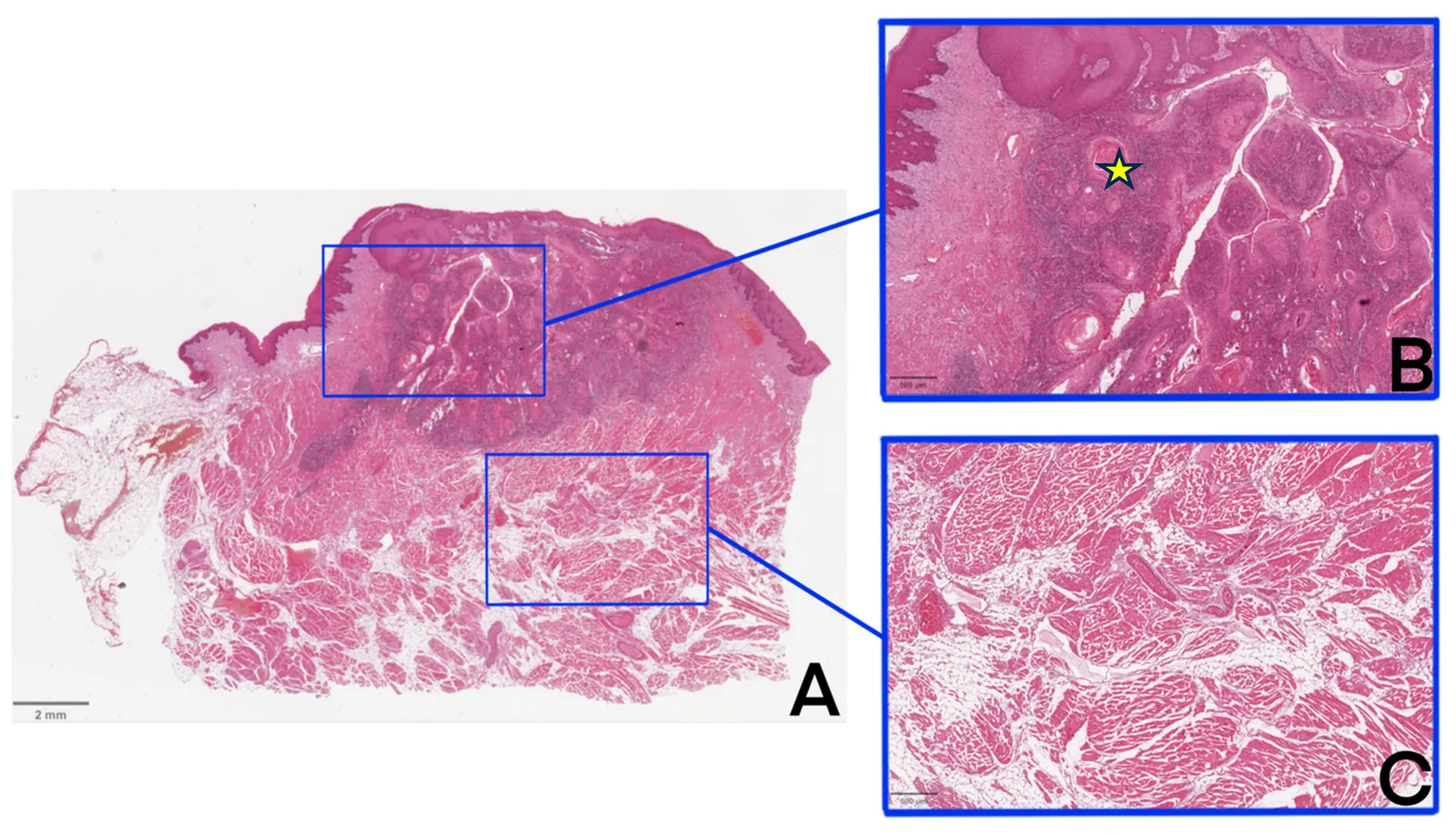
Patient A., female, 62 years. Histological section of tongue OSCC; H&E stain. (**A**) Low magnification view showing the tumor mass (top) and underlying muscle tissue (bottom); (**B**) Invasive squamous cell carcinoma with a prominent keratin pearl (★); (**C**) Normal skeletal muscle tissue of the tongue with preserved striation.

**Figure 2:**
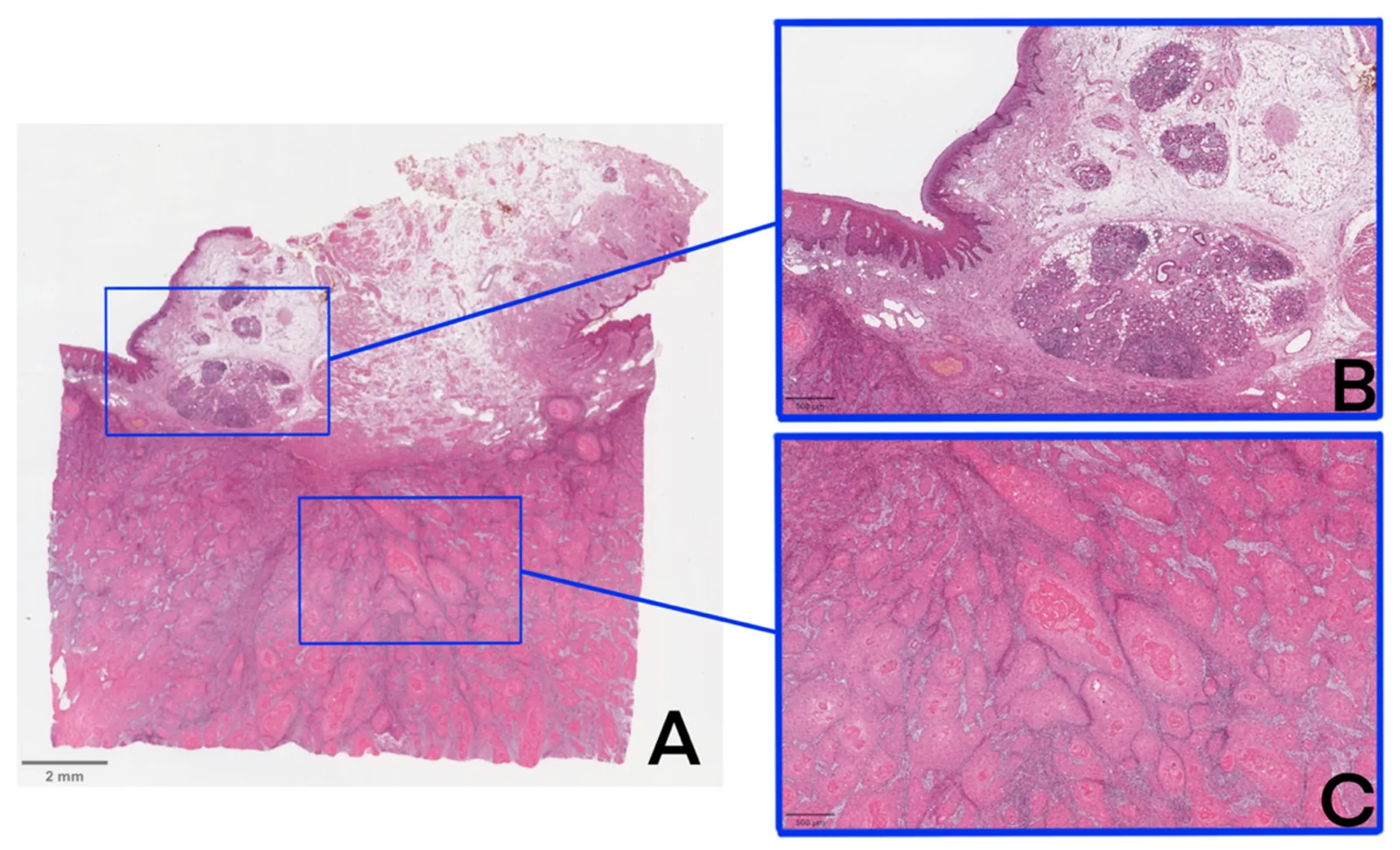
Patient A., female, 50 years. Histological section of lower lip biopsy; H&E stain. (**A**) Low-magnification view showing tumor mass below the normal mucosa. (**B**) Normal oral mucosa with minor salivary glands. (**C**) Invasive squamous cell carcinoma with prominent keratin pearls and cellular atypia.

All tumor samples demonstrated histopathological features consistent with squamous cell carcinoma. The neoplastic tissue was composed of invasive nests, cords, and sheets of malignant squamous epithelial cells infiltrating the underlying connective tissue. Tumor cells exhibited marked cytological atypia, including eosinophilic cytoplasm, hyperchromatic nuclei, nuclear and cellular pleomorphism, and increased mitotic activity. Intercellular bridges and individual cell keratinization were frequently observed. In well-differentiated areas, keratin pearl formation was present. The tumor stroma was variably fibrotic and often showed peritumoral and intratumoral inflammatory infiltrates composed predominantly of lymphocytes and histiocytes. Focal edema, vascular congestion, and areas of necrosis were identified in some samples, particularly in more advanced or deeply invasive tumors. Mitotic figures, including atypical mitoses, were readily identifiable, with mitotic activity increasing with tumor grade and depth of invasion. Early invasive lesions demonstrated superficial stromal infiltration, whereas advanced tumors showed extensive invasion into deeper connective tissue structures. Squamous cell carcinoma is characterized by limited superficial invasion confined to the primary tissue without regional spread, whereas stage II tumors demonstrate greater depth of invasion and increased tumor size, reflecting more aggressive local growth. No fundamental histological differences were found between squamous cell carcinoma of the tongue and lip (Figure 1 and Figure 2).

### 3.3. Immunohistochemical Assay

**Figure 3:**
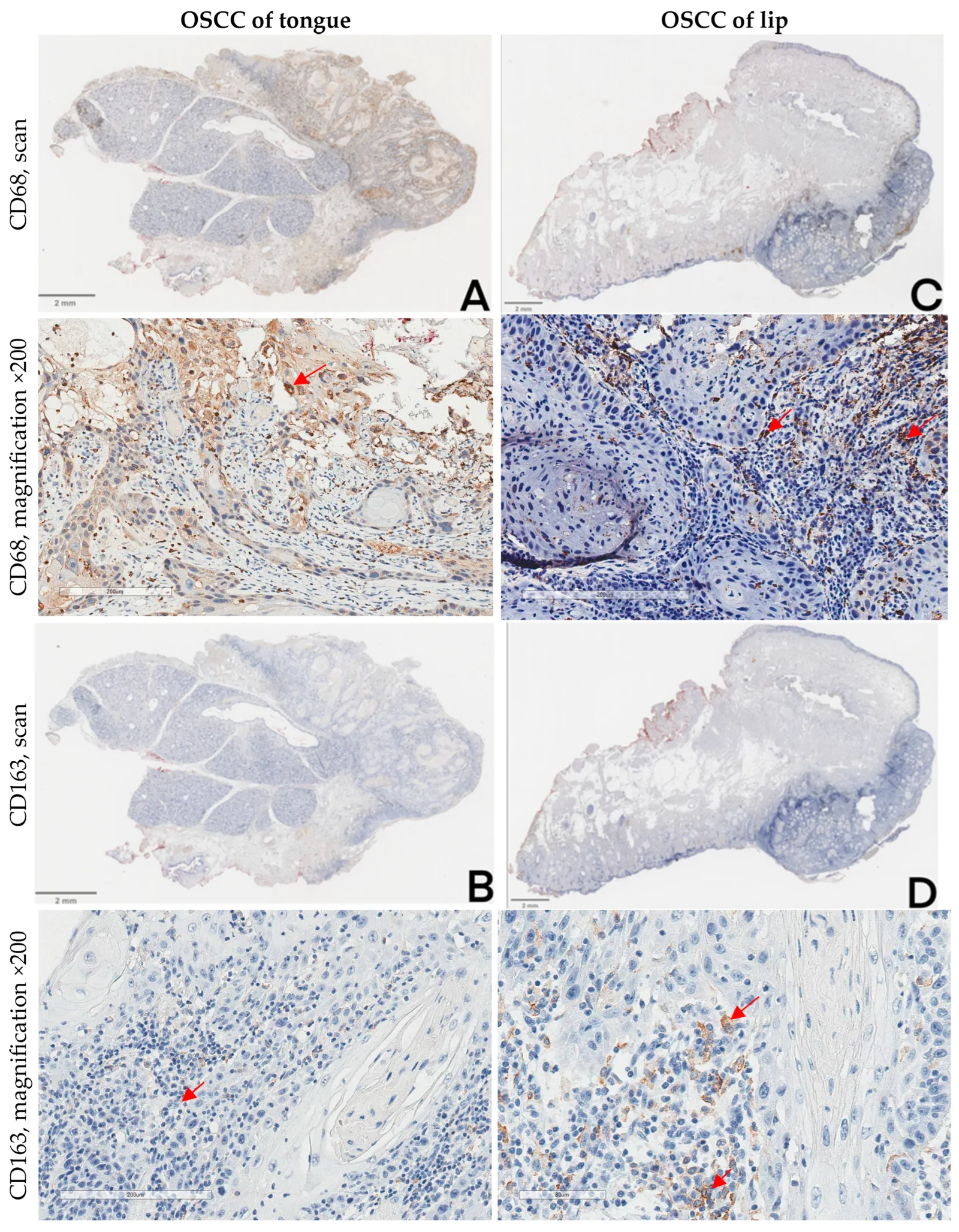
Immunohistochemical staining with antibodies to CD68 and CD163 macrophages (arrows) in tongue and lip OSCC; magn. ×200. (**A**) Patient V., male, 53 years; (**B**) Patient V., male, 53 years; (**C**) Patient A., female, 61 years; (**D**) Patient A., female, 61 years.

Immunohistochemical staining revealed the presence of CD68^+^ and CD163^+^ macrophages in both intratumoral and peritumoral compartments of oral squamous cell carcinoma of the tongue and lip. Tumor-associated macrophages (TAMs) were predominantly observed as scattered single cells or small clusters, with a tendency to accumulate in areas of dense tumor cell infiltration and active stromal remodeling. CD68^+^ macrophages exhibited moderate to strong cytoplasmic immunoreactivity and demonstrated variable cellular morphology with oval to polygonal nuclei and occasional irregular nuclear contours. In contrast, CD163^+^ macrophages displayed a more uniform, rounded to spindle-shaped morphology, with distinct membrane-associated staining and smaller, more homogeneous nuclei. Macrophage infiltration was detected both within the tumor parenchyma (intratumoral localization) and in the surrounding stromal tissue (peritumoral localization). The peritumoral regions frequently demonstrated a higher density of CD163^+^ macrophages, whereas CD68^+^ cells were more evenly distributed between intratumoral and peritumoral compartments. In the adjacent non-tumorous oral mucosa, only sparse CD68^+^ macrophages were identified within the lamina propria, primarily in association with vascular structures. CD163^+^ macrophages in these areas were rare or absent (Figure 3).

### 3.4. Distribution of CD68^+^ and CD163^+^ Macrophages in OSCC

**Figure 4:**
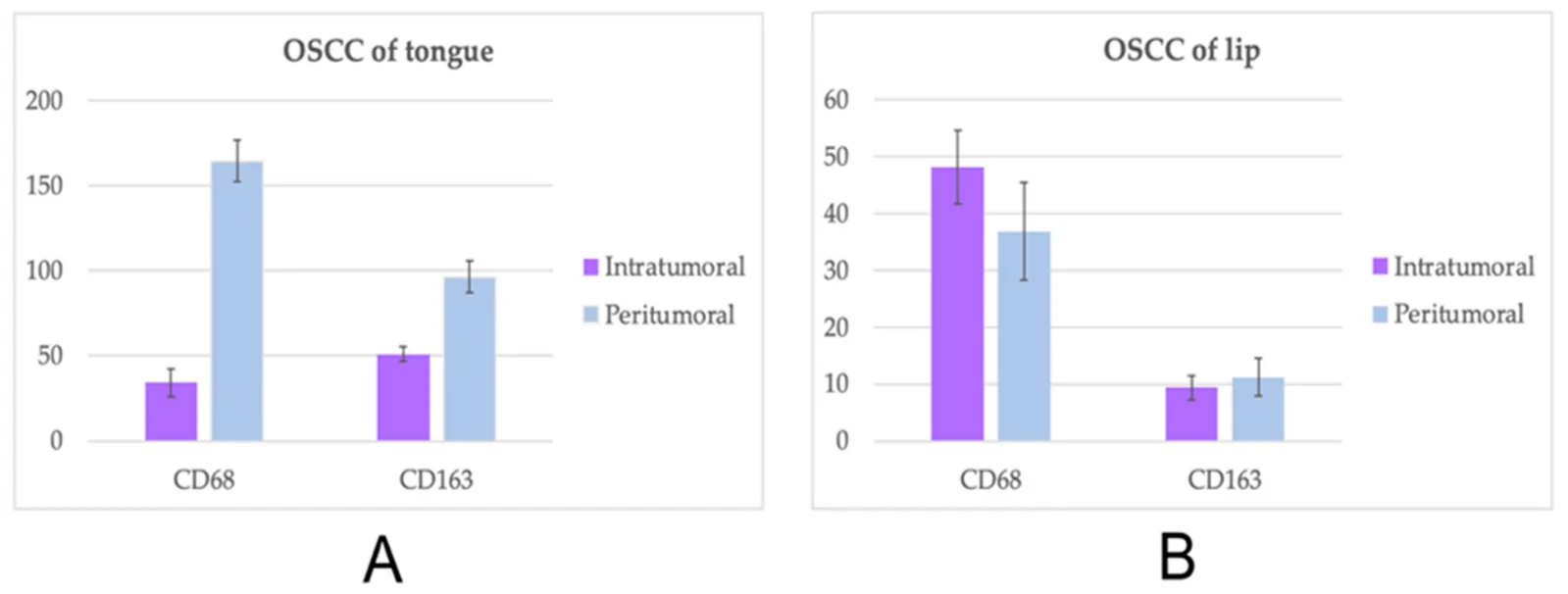
Diagrams showing the number of macrophages CD-68 and CD-163 intratumorally and peritumorally: (**A**) OSCC of tongue, (**B**) OSCC of lip.

In OSCC of the tongue, CD68^+^ tumor-associated macrophages, representing the overall macrophage population, were detected both within the tumor nests and in the surrounding tumor microenvironment. Quantitative immunohistochemical analysis demonstrated a significantly higher density of CD68^+^ cells in the peritumoral compartment than within the tumor nests (164.4 ± 12.3 vs. 34.2 ± 8.1 cells/mm^2^), indicating preferential accumulation of macrophages in the stromal microenvironment surrounding the tumor. Similarly, CD163^+^ macrophages showed greater infiltration in the peritumoral microenvironment compared with the intratumoral compartment (96.5 ± 9.1 vs. 51.1 ± 4.3 cells/mm^2^), suggesting enrichment of M2-polarized macrophages in the stroma adjacent to the tumor nests. In contrast, OSCC of the lip demonstrated a different pattern of macrophage distribution. CD68^+^ macrophages were more abundant within the tumor nests than in the peritumoral compartment (48.1 ± 6.4 vs. 36.8 ± 8.6 cells/mm^2^), indicating a predominance of intratumoral macrophage localization. CD163^+^ macrophages were virtually absent in both the intratumoral and peritumoral compartments, with density values of 9.4 ± 2.1 cells/mm^2^ within the tumor nests and 11.2 ± 3.3 cells/mm^2^ in the peritumoral stroma, remaining consistently below 10 cells/mm^2^ in the tumor microenvironment and confirming the lack of M2 polarization in this lip OSCC cohort (Figure 4).

## 4. Discussion

The present study assessed the spatial distribution of CD68^+^ and CD163^+^ macrophages in OSCC of the tongue and lip. The most striking finding was the active accumulation of both CD68^+^ and CD163^+^ macrophages in the peritumoral stroma of tongue OSCC, whereas lip OSCC demonstrated a markedly lower macrophage density and a near-complete absence of CD163^+^ cells in both tumor compartments (intratumoral and peritumoral). These observations point to substantial differences in the immune microenvironment between the two anatomical sites and suggest that macrophage polarization may contribute to the biological heterogeneity of OSCC [14].

**Figure 5:**
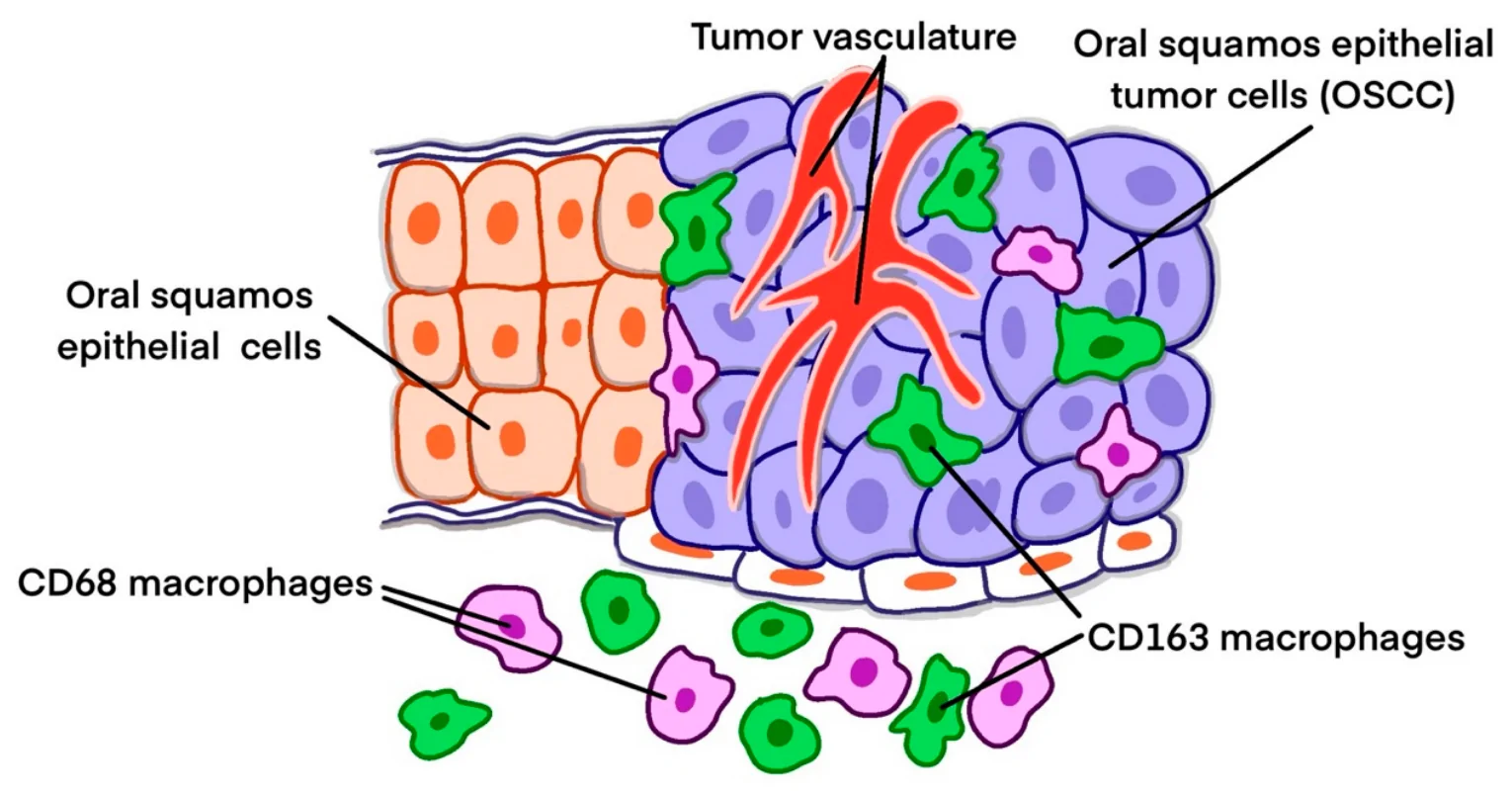
Ratio of CD68^+^ and CD163^+^ macrophages in OSCC. Schematic representation, original illustration.

One of the key findings of the present study was the predominant localization of CD68^+^ macrophages in the peritumoral stroma of tongue OSCC. The peritumoral compartment represents a dynamic zone of interaction between malignant epithelial cells and the adjacent connective tissue and is considered a critical region for tumor invasion and progression. As reported by Xue et al. [15], TAMs frequently accumulate at the invasive tumor front, where they participate in extracellular matrix remodeling, angiogenesis, and tumor cell migration (Figure 5).

At the molecular level, this recruitment is driven chiefly by tumor cell– and fibroblast-derived colony-stimulating factor-1 (CSF-1/M-CSF) and the chemokine CCL2 (MCP-1), which engage their cognate receptors CSF1R and CCR2 on circulating monocytes and direct their transendothelial migration toward the invasive margin [16]. Once resident in the stroma, these macrophages secrete matrix metalloproteinase-9 (MMP-9) and MMP-2, which degrade type IV collagen of the basement membrane and remodel the peritumoral extracellular matrix, while concomitant secretion of vascular endothelial growth factor-A (VEGF-A), acting through VEGFR2 on endothelial cells, promotes local neoangiogenesis [17]. Together, these two processes lower the physical barrier to invasion and supply the metabolic support required for tumor cell egress into the surrounding stroma.

Suarez-Sanchez et al. [18] reported that increased TAM infiltration is associated with epithelial–mesenchymal transition and unfavorable clinicopathological characteristics of OSCC, highlighting the importance of macrophage localization within the tumor microenvironment. Similar observations are discussed in a review by Bashah et al. [19], whose authors note that macrophage-rich stromal areas are consistently associated with a more aggressive disease course and an unfavorable prognosis. Mechanistically, TAM-derived transforming growth factor-β1 (TGF-β1) signals through the type II TGF-β receptor (TβRII) and activates SMAD2/SMAD3, which in turn induce the transcriptional repressors of E-cadherin (Snail (SNAI1), Slug (SNAI2), ZEB1 and Twist1) in adjacent carcinoma cells. This program produces the loss of membranous E-cadherin and the gain of vimentin and N-cadherin that characterize EMT at the invasive front [13]. Notably, Twist1-driven tumor cells have themselves been shown to upregulate CSF-1, creating a feed-forward loop in which EMT reinforces macrophage recruitment and recruited macrophages, in turn, sustain EMT signaling—a plausible molecular substrate for the co-localization of CD68^+^ TAMs with invasive tumor budding observed in our tongue OSCC cohort [20].

Another important observation was the significant presence of CD163^+^ macrophages in the peritumoral stroma of tongue OSCC. CD163 is widely regarded as a marker of M2-polarized macrophages, which predominantly exert immunosuppressive and tumor-promoting functions. Structurally, CD163 is a class B scavenger receptor of the scavenger receptor cysteine-rich (SRCR) superfamily that mediates endocytic clearance of haptoglobin–hemoglobin complexes; its ligation triggers heme oxygenase-1 (HO-1) induction and interleukin-10 (IL-10) secretion, reinforcing an anti-inflammatory, tissue-remodeling macrophage phenotype [21,22]. CD163 expression is transcriptionally driven by the canonical M2-polarizing cytokines IL-4, IL-13 and IL-10 acting through STAT6, and is potentiated by sustained CSF-1/CSF1R signaling; within hypoxic tumor stroma, stabilization of hypoxia-inducible factor-1α (HIF-1α) further skews newly recruited monocytes toward this CD163^high phenotype [21]. In the present study, CD163^+^ cells exhibited a distribution pattern similar to that of the total macrophage population but were most pronounced in the stromal component surrounding the tumor growth zones. These findings are consistent with the data of Troiano et al. [23], who identified a high density of CD163^+^ macrophages in invasive stromal areas of OSCC and demonstrated an independent association between elevated CD163 expression and unfavorable disease prognosis. Similarly, Bisheshar et al. [24] demonstrated that increased CD163 expression correlates with metastasis to regional lymph nodes and reduced overall survival in OSCC patients. This clinical association has a direct immunological correlate: CD163^+^ TAMs frequently co-express the immune checkpoint ligand PD-L1 and secrete IL-10, both of which suppress cytotoxic CD8^+^ T-cell activity and dampen antitumor immunosurveillance, providing a mechanistic basis for the poor outcomes reported in CD163-high tumors [25,26]. Collectively, these data suggest that the macrophage phenotype may be of greater clinical significance than the absolute macrophage count.

The present study also revealed pronounced differences between OSCC of the tongue and lip. Whereas tongue tumors were characterized by substantial infiltration with CD163^+^ cells, CD163^+^ macrophages were virtually absent in lip tumors, despite the presence of CD68^+^ macrophages. This observation suggests that macrophage recruitment and their subsequent polarization represent distinct biological processes. Tongue OSCC is known to be characterized by a more aggressive clinical course, a higher rate of regional metastasis, and a greater propensity for local invasion compared with tumors of other oral cavity subsites, including the vermilion border of the lip [27]. Moreover, the characteristics of the immune microenvironment are regarded as one of the key factors determining the biological behavior of different OSCC forms and the composition of infiltrating immune cells [28]. A plausible molecular explanation for this dissociation lies in the distinct signaling requirements for monocyte recruitment versus polarization. Recruitment via the CSF-1/CSF1R and CCL2/CCR2 axes appears to proceed comparably in both tumor types. Polarization toward the M2 phenotype, however, requires additional and more sustained signals—prolonged exposure to TGF-β1 and IL-10, together with stabilization of HIF-1α under hypoxic conditions [22,29]. These polarizing signals appear less pronounced in the more indolent, superficially growing lip carcinomas than in the deeply invasive tongue tumors, which are more likely to generate the hypoxic, metabolically demanding niches that favor CD163^high polarization [16,29]. Consequently, the pronounced accumulation of CD163^+^ macrophages in tongue tumors may reflect the formation of a more aggressive and immunosuppressive microenvironment.

The present study also notes a discrepancy between CD68 and CD163 expression levels. Whereas CD68^+^ macrophages were identified in both tumor types, CD163^+^ macrophages were predominantly detected in tongue OSCC. The findings obtained suggest that assessing total macrophage density alone may not accurately reflect the biological activity of the tumor microenvironment. Several studies have shown that CD163 is a more reliable prognostic marker than CD68, as CD68 identifies a heterogeneous macrophage population comprising both tumor-promoting and potentially tumor-suppressive subpopulations. This heterogeneity has a molecular basis. CD68^+^ macrophages encompass cells along the entire polarization spectrum. At one end are classically activated, antitumor M1 macrophages, which depend on STAT1 and interferon regulatory factor 5 (IRF5) signaling downstream of interferon-γ and produce inducible nitric oxide synthase (iNOS), TNF-α, and IL-12 [30]. At the other end are alternatively activated, tumor-promoting M2 cells, identified primarily by CD163 and, to a lesser extent, by CD206 (MRC1) [31]. In particular, a meta-analysis by Hadler-Olsen and Wirsing [32], as well as a more recent meta-analysis by Chohan et al. [33], demonstrated a stronger association of CD163^+^ TAMs with unfavorable prognosis compared with the general CD68^+^ macrophage population. Consequently, phenotypic characterization of TAMs may provide more meaningful information on the biological behavior of a tumor than quantitative assessment of the total macrophage population.

Taken together, our findings indicate that oral squamous cell carcinoma engages a coordinated, site-dependent molecular program to remodel its macrophage compartment. Tumor-derived CSF-1 and CCL2 recruit circulating monocytes to the peritumoral stroma; TGF-β1/SMAD3 and IL-6/STAT3 signaling subsequently drive both epithelial–mesenchymal transition in adjacent carcinoma cells and progressive polarization of resident macrophages toward the CD163^+^, HIF-1α-stabilized, PD-L1-associated M2 phenotype; and macrophage-derived MMP-9, MMP-2 and VEGF-A complete this circuit by remodeling the extracellular matrix and supporting angiogenesis at the invasive front [34]. This cascade appears substantially more active in tongue carcinoma, which may account for its more aggressive clinical behavior relative to lip carcinoma, and it underscores the potential of the CSF-1/CSF1R and CCL2/CCR2 axes, alongside CD163 itself, as candidate targets for microenvironment-directed adjuvant strategies in OSCC.

### Limitations

This study is limited by its retrospective design, the two-marker immunophenotypic panel (CD68/CD163), and the absence of patient outcome data, including direct correlation between macrophage phenotype and prognosis within this cohort. The analysis of clinicopathological and behavioral risk factors (such as alcohol use or exposure to tobacco smoke) was not the objective of the present retrospective analysis. In light of the foregoing, future prospective studies incorporating additional polarization markers (e.g., CD206, iNOS) or multiplex spatial and single-cell approaches, together with long-term survival follow-up and systematic collection of clinicopathological and lifestyle risk-factor data, would help clarify the prognostic value of site-specific macrophage polarization in OSCC; mechanistic validation of the CSF-1/CSF1R and CCL2/CCR2 recruitment axes could further inform microenvironment-directed adjuvant strategies, particularly for the more aggressive, CD163-enriched tongue OSCC phenotype.

## 5. Conclusions

The present study revealed pronounced differences in distribution and polarization of CD68^+^ and CD163^+^ macrophages between OSCC of the tongue and lip. Tongue OSCC was characterized by the predominant accumulation of both CD68^+^ and CD163^+^ macrophages in the peritumoral stroma compared with the intratumoral zones. In contrast, in lip OSCC, a higher density of CD68^+^ macrophages was observed in the intra-tumoral compartment, whereas the number of CD163^+^ macrophages remained considerably lower in both the intratumoral and peritumoral stroma of the tumor. These findings underscore the role of the immune microenvironment in the biological heterogeneity of OSCC and warrant further investigation into TAMs depending on the anatomical location in the oral cavity, which is very important when choosing antitumor therapy and surgical treatment.

## Figures and Tables

**Table 1:** Clinicopathological characteristics of the patients.

Characteristic	Localisation	Rate
Age	Tongue	29–88 years
	Lip	
Average age	Tongue	64.2 ± 6.8 years
	Lip	
Male	Tongue	58
	Lip	19
Female	Tongue	26
	Lip	9
Type of OSCC		
Squamous Cell Carcinoma of tongue	84	
Squamous Cell Carcinoma of lip	28	
pT1	112	

## Data Availability

The original contributions presented in this study are included in the article. Further inquiries can be directed to the corresponding author.
